# Multilamellar Structures and Filament Bundles Are Found on the Cell Surface during Bunyavirus Egress

**DOI:** 10.1371/journal.pone.0065526

**Published:** 2013-06-14

**Authors:** Laura Sanz-Sánchez, Cristina Risco

**Affiliations:** Cell Structure Laboratory, Centro Nacional de Biotecnología, CNB-CSIC, Campus de Cantoblanco, Madrid, Spain; University of the Witwatersrand, South Africa

## Abstract

Inside cells, viruses build specialized compartments for replication and morphogenesis. We observed that virus release associates with specific structures found on the surface of mammalian cells. Cultured adherent cells were infected with a bunyavirus and processed for oriented sectioning and transmission electron microscopy. Imaging of cell basal regions showed sophisticated multilamellar structures (MLS) and extracellular filament bundles with attached viruses. Correlative light and electron microscopy confirmed that both MLS and filaments proliferated during the maximum egress of new viruses. MLS dimensions and structure were reminiscent of those reported for the nanostructures on gecko fingertips, which are responsible for the extraordinary attachment capacity of these lizards. As infected cells with MLS were more resistant to detachment than control cells, we propose an adhesive function for these structures, which would compensate for the loss of adherence during release of new virus progeny.

## Introduction

Viruses manipulate cell organization by recruiting materials to build scaffolds, where they replicate their genomes, assemble new infectious particles, and conceal themselves from antiviral defense sentinels of the cell [1],[2]. These virus activities damage the cell, which can respond with self-defensive structural solutions such as specialized cytosolic or nuclear bodies in which viral factors are trapped and immobilized [3],[4]. Although some viruses are degraded in autophagosomes and aggresomes, some others can in fact induce and use these organelles to build their replication sites [5].

Virus-induced intracellular compartments have been the subject of numerous studies using light and electron microscopy. In addition, viruses enter the cell through plasma membrane structures; the membrane is the first barrier viruses must overcome to infect a cell, and the last when they are ready for egress and propagation. Virus entry is most commonly associated with caveolae, clathrin-coated vesicles, or filopodia; these last assist virus entry during macropinocytosis [6], [7]. Viruses leave cells by active secretion, cell lysis, or with the assistance of virus-induced structures assembled on the cell surface such as actin comets, viral synapses, filopodia or nanotubes [8]–[12]. The specific surface used for egress varies with virus and cell type; in adherent cultured cells, viruses can exit through the basal, apical or basolateral cell surfaces [13]–[16]. Directed release might affect virus invasive capacity in certain tissues, as well as its propagation within the organism [17]–[19]. To characterize and understand the structural solutions that arise from this virus-cell crosstalk, live cell video microscopy and correlative light and electron microscopy (CLEM) provide new ways to examine cell processes and structures that have been overlooked using conventional approaches [3], [20]. CLEM allows pre-selection of individual live cells with features of interest, for detailed ultrastructural study in transmission electron microscopy (TEM). With these powerful tools, we can analyze complex events in heterogeneous cell populations and address the biogenesis and evolution of cell structures such as those induced by virus infection [1], [21].

We previously reported that Bunyamwera virus (BUNV), the best characterized member of the family *Bunyaviridae*, transforms cell structure to build an intracellular factory that changes according to virus needs during the phases of genome replication and morphogenesis [22]. The key element of BUNV factories is the Golgi complex, where the virus collects the replication complexes, and new viral particles assemble and mature [22], [23]. At the *trans*-Golgi network, viruses are gathered inside secretory vesicles and transported to the cell periphery; they finally exit the cell by normal secretion [24]. Here we studied the structural transformations of the cell during the last phase of the virus life cycle, virus egress and propagation. Oriented serial sections and TEM showed two unreported virus-induced structures on the basal surface of BUNV-infected cells. CLEM showed that complex multilamellar structures (MLS) and extracellular bundles of filaments with many attached viruses proliferated during the phase of maximum virus egress. MLS assembled in cells that release new virus progeny through the basal surface, but were not found in those that release viruses through the apical surface. MLS morphology and dimensions implied a role in cell attachment, a possibility supported by functional adhesion tests. The effects of cytochalasin D in infected cells suggested that the extracellular actin filament bundles participate in virus release and possibly in virus propagation to neighboring cells.

## Results and Discussion

### Live cell video microscopy, CLEM and TEM of control and bunyavirus-infected cells

**Figure 1:**
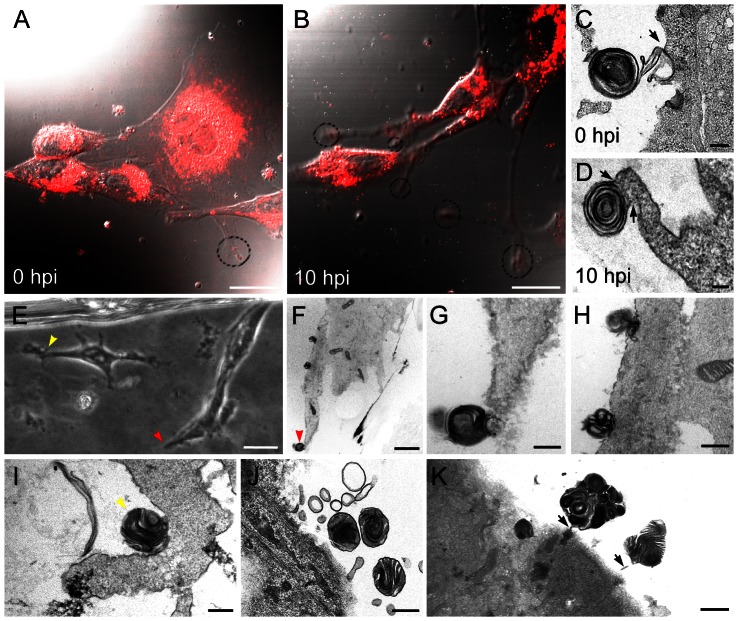
Live cell video microscopy, TEM and CLEM of mock- and bunyavirus-infected BHK-21 cells. (A, B) Differential interference contrast and WGA-Alexa594 fluorescence microscopy (red) of mock- (A) and BUNV-infected (B) cells at 10 h.p.i. Long projections (dashed circles) proliferate in infected cells. (C, D) TEM of ultrathin sections of basal regions of mock- and BUNV-infected cells, showing round lamellar structures connected with cell surface projections. (E–I) CLEM of infected cells. (E) Phase contrast light microscopy of two infected cells with long projections (yellow and red arrowheads). TEM of the cell on the right is shown in (F); (G, H) high-magnification views of the three multilamellar structures (MLS) in (F). (I) TEM of the cell projection marked with a yellow arrowhead in (E) showing an MLS. (J, K) Several round MLS from different cells. Bars: 25 µm (A and B), 200 nm (C), 100 nm (D), 10 µm (E), 2 µm (F), 400 nm (G and H), 300 nm (I), 500 nm (J), 1 µm (K).

BHK-21 cells were infected with BUNV at a multiplicity of infection (M.O.I.) of 1 plaque-forming unit (PFU) per cell, and live cells were studied by video microscopy (Figure 1). Infected cells progressively developed long projections, which were more numerous at 8–10 h post-infection (h.p.i.) as seen by differential interference contrast (DIC) imaging and confocal microscopy (Figure 1A, B). At 10 h.p.i., all cells in the monolayer are infected; this time post-infection coincides with maximum release of new infectious viruses, as shown by time-course studies [23], [24]. Projections developed long before infected cells deteriorated and died; these projections are not associated to cell contraction prior to cell division, because mitosis is inhibited in BUNV-infected mammalian cells [3], [22]–[24]. BHK-21 cells released new viruses through the surface in contact with the culture plate ([22] and see below). Although these cells are not polarized, we will refer to this surface as “basal”, whereas the external surface far from the culture plate will be termed “apical”. Independent observations of BHK-21 cells processed by oriented sectioning and TEM (described in Figure S1) showed that the basal regions of control and infected cells bore MLS (Figure 1C, D), which were more numerous in infected than in control cells: on average, 2–3 vs 0–1 MLS per cell, respectively (*n* = 900). The presence of MLS in control cells was rare. To determine whether MLS were associated with the projections that proliferated in infected cells as observed by live cell video microscopy (Figure 1B), we used CLEM to study cell cultures (Figure 1E–I). Using light microscopy, we selected live cells with long projections for oriented serial sectioning and electron microscopy (Figure 1E). Ultrastructural analysis confirmed MLS association with long projections (Figure 1F–I); most MLS were rounded, were ∼500–600 nm in diameter, and showed characteristic packed lamellae (Figure 1J, K). In CLEM studies, the first images generated by light microscopy show whole cells. For subsequent TEM analysis, we prepare serial thin sections, which are single planes of these cells (since this can produce small differences in cell contours, we use red and yellow arrowheads to facilitate correlation (Figure 1E, F)).

Similar MLS were detected in basal surfaces of BUNV-infected human MRC-5 fibroblasts (not shown); massive virus release also takes place through the basal region of these cells (see below). The ultrastructure of MLS was best preserved when monolayers were fixed without removing cell culture medium and by avoiding extensive washing before fixation.

**Figure 2:**
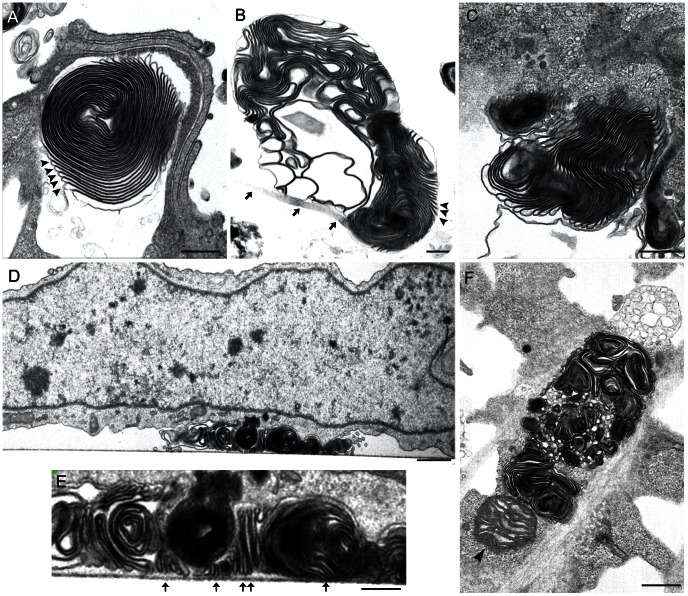
Ultrastructural details of complex MLS in BUNV-infected BHK-21 cells. (A–C, F) MLS in sections oriented parallel to and (D, E) MLS in sections oriented perpendicular to the cell base. (A) MLS with numerous densely packed, regularly spaced lamellae (arrowheads). (B) Another MLS with regularly spaced lamellae (arrowheads) and a thin connection with the cell surface (arrows). (C) Complex MLS with many lamellae projecting towards the cytosol; no obvious contacts with intracellular membranes are seen. (D) MLS attached to the culture plate as seen in a section oriented perpendicular to the cell base. (E) Enlargement of the MLS in (D), showing lamellar contact with the culture plate (arrows). (F) Complex MLS with many lamellae and a nearby mitochondrion (arrowhead). Bars: 400 nm (A, B and C), 1 µm (D), 300 nm (E), 600 nm (F).

After confirming MLS association with infection, we performed a detailed ultrastructural study. Infected cell monolayers were processed by serial sectioning and TEM. Sections were oriented parallel or perpendicular to the cell base (Figure 2, S1). Together with small, simple MLS (Figure 1), larger, more complex MLS were frequently detected in BUNV-infected BHK-21 and MRC-5 cells, but not in uninfected controls. MLS were composed of closely packed lamellae of regular thickness and spacing (Figure 2A–C). In sections oriented perpendicular to the cell base, lamellae contacted the culture plate (Figure 2D); some attached to the base at a 90° angle, while others varied in angle and orientation (Figure 2E).

Lamellar dimensions and morphology, as well as their mode of interaction with the substrate, was reminiscent of the super-adhesive structures on gecko fingertips [25]. The smallest subdivisions of the fingertips of these lizards are the spatula tips, which have been studied by scanning electron microscopy and atomic force microscopy. Their dimensions (∼500×200–300×10 nm; length×width×thickness) [26]–[29] show clear similarity to MLS size (∼600×∼30×∼20 nm) (Figure 2). No sticky substances appear to participate in this mechanical function of the tips, whose adhesive role is based on structural design and van der Waals forces [25].

Stringent analysis of more than 500 MLS revealed no obvious connections between these structures and intracellular elements; for example, mitochondria were often seen near MLS, but no clear contacts were detected (Figure 2F). Serial sections and 3D reconstructions showed that MLS exclusively contacted the plasma membrane (Figure S2). The lamellar structure of MLS was reminiscent of cell lysosomes. To determine whether MLS are related to secretory lysosomes, we studied the distribution of these organelles in control and BUNV-infected cells. Lysotracker, a marker of acidic compartments such as lysosomes, was concentrated at the perinuclear region of control and BUNV-infected BHK-21 cells (Figure S3). We detected no organelle movement towards the basal adherent surface or the cell periphery during the course of infection. Results were also negative for control and BUNV-infected MRC-5 cells labeled with antibodies specific for the lysosome transmembrane protein Lamp-1 or for CD63, a marker of late endosomes, multivesicular bodies and lysosomes (not shown); moreover, we were unable to obtain any images of lysosome secretion events. We conclude that virus-induced MLS do not originate in secretory lysosomes.

**Figure 3:**
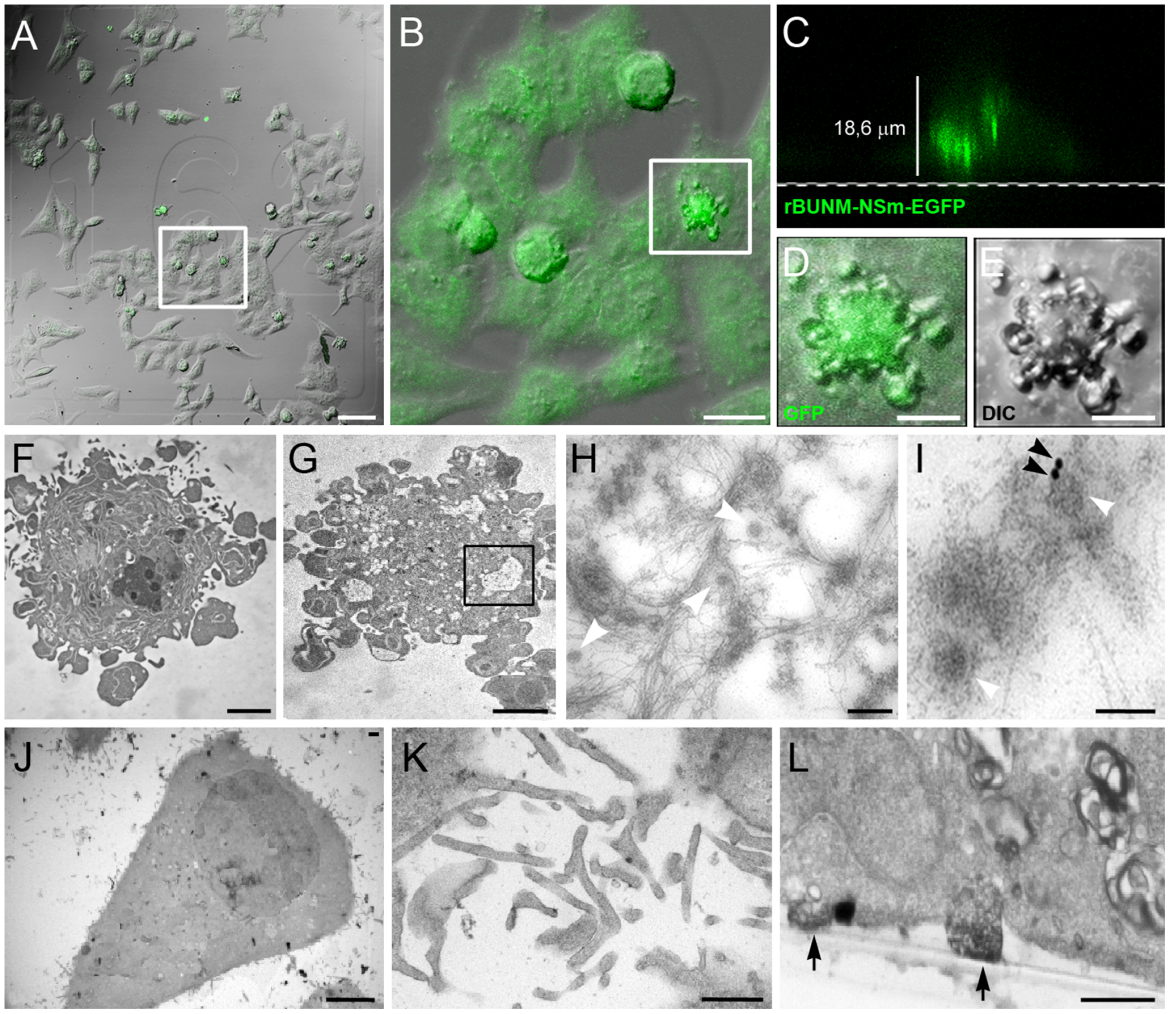
TEM and CLEM of control and BUNV-infected HEp-2 cells. (A, B) Differential interference contrast (DIC) and GFP-fluorescence microscopy (frontal projections) of HEp-2 cells infected with recombinant rBUNM-NSm-EGFP virus at 30 h.p.i. (A) The group of cells selected for CLEM and marked with a white rectangle is enlarged in (B). (C) Lateral merge projection of the cell inside the white square in (B) as visualized by confocal microscopy. The green signal is detected in mid- to upper regions of the cell, showing virus assembly sites. (D, E) Enlargement of the selected cell visualized by GFP-fluorescence microscopy and DIC, respectively (frontal projection). (F, G) TEM of oriented ultrathin serial sections of the selected cell. Sections are oriented parallel to the culture plate and obtained at 11 µm (F) and 29 µm (G) from the cell base. (H) Enlargement of the area labeled with a black rectangle in (G), showing viral particles (white arrowheads) surrounded by filaments on the upper cell surface. (I) Immunogold labeling of viral particles with an antiserum to the viral nucleocapsid protein (N) and secondary antibodies conjugated with 15 nm colloidal gold particles (black arrowheads). (J, K) TEM of control HEp-2 cells. (J) Low magnification showing the characteristic shape of a healthy control HEp-2 cell. (K) Ultrathin section of basal regions with numerous filopodia. (L) TEM of infected HEp-2 cells at 48 h.p.i; this section is oriented perpendicular to the cell base. The cell on the left is attached to the culture plate with podosome-like structures (arrows). Bars: 75 µm (A), 25 µm (B), 10 µm (D and E), 3 µm (F and G), 200 nm (H), 100 nm (I), 6 µm (J), 600 nm (K and L).

BUNV-infected BHK-21 and MRC-5 cells are hamster and human fibroblasts, respectively, which release new virus progeny through the cell basal regions. We tested whether MLS also assemble in cells in which the virus is not released through basal surfaces. For these experiments, we used recombinant BUNV encoding the NSm viral scaffolding protein fused to GFP [30] (Figure 3A); this protein concentrates in the BUNV assembly compartment and is not incorporated into mature virions [22]. Infected HEp-2 human cells were processed by CLEM and oriented for serial sectioning (Figure 3). Time-course experiments showed that in cultured monolayers of BUNV-infected HEp-2 cells the maximum release of new infectious viruses is reached at 26–30 h.p.i. (not shown). Cells with strong fluorescent signals in confocal microscopy (Figure 3A, B) were selected and processed sequentially by CLEM, oriented serial sectioning, and TEM. Lateral merge images showed that fluorescence signals that indicate virus assembly sites were concentrated at different levels within the cells (Figure 3C). After processing selected cells by serial sectioning and TEM, we found that viral particles were released exclusively through apical cell surfaces (Figure 3F–I), where viruses were frequently surrounded by thin filaments (Figure 3H, I). A search for specialized structures in HEp-2 cell basal regions showed abundant filopodia, but no MLS (Figure 3J, K). Sections oriented perpendicular to the cell base showed podosome-like structures (Figure 3L), but again no MLS. In HEp-2 cells, in which virus is released through apical regions, no MLS appear to assemble.

### Resistance to detachment by control and BUNV-infected cells

**Figure 4:**
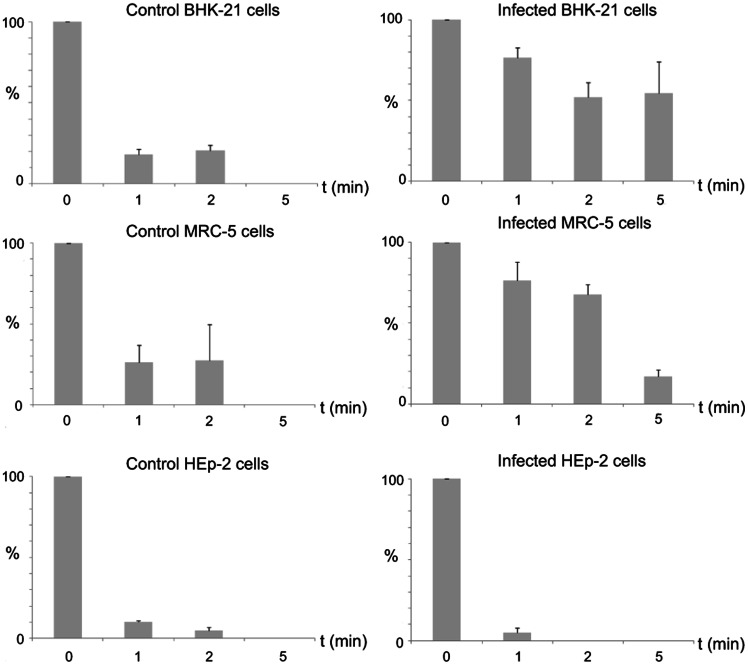
Resistance of control and BUNV-infected cells to detachment induced by incubation with trypsin-EDTA. Histograms summarize the results of three independent experiments and show the percentage of cells that remained attached to the culture plates after incubation with trypsin-EDTA for 1, 2 or 5 min. Each bar represents mean ± SEM (*n* = 3). Times post-infection correspond to the phase of maximum virus release (10 h.p.i. for BHK-21, 16 h.p.i. for MRC-5 cells and 26 h.p.i. for HEp-2 cells). At the indicated t.p.i and a M.O.I. of 1 PFU/cell, all cells in the monolayers were infected.

MLS morphology and dimensions suggested a role in cell attachment. To determine whether the presence of MLS alters cell capacity to attach to culture plates, we studied the response of monolayers with and without MLS incubated with trypsin-EDTA for 1, 2 and 5 min. This treatment is widely used to detach adherent cells from culture plates before subculture. Cells that remained attached to plates after trypsin-EDTA addition were quantified. We found that cells with few or no MLS (control BHK-21, control MRC-5 cells, as well as control and infected HEp-2) detached rapidly. Cells with more numerous, complex MLS (infected BHK-21 and MRC-5 cells) were more resistant to trypsin-EDTA incubation and remained attached for longer times. After 10 min incubation with trypsin-EDTA, all cells had detached from plates. Data for three independent experiments are summarized in Figure 4.

These observations suggest that a structure similar to that which allows adhesion in complex organisms such as geckos might have a comparable role in mammalian cells. Gecko-based morphology is being used to design industrial devices [31]–[33]. These biomimicry studies seek functional designs in nature to create new machines and products; for example, the super-adhesive capacity of gecko fingertip structure was used in robots able to adhere to and walk on smooth vertical and inverted surfaces [34]. In virus-infected mammalian cells, MLS attachment capacity could be a response to evade detachment and death. We observed partial cell detachment from the substrate during massive release of virus progeny through basal regions. Cells might sense detachment and trigger a signal for MLS assembly. Mammalian cells have plasma membrane reservoirs that allow rapid assembly of filopodia, lamellipodia, blebs and pseudopodia [35]–[37]; MLS might also be assembled from these surface membrane stocks. It remains to be determined whether changes in protein levels and/or intracellular distribution involved in plasma membrane dynamics and cell adhesion can trigger a specific MLS assembly signal. Our preliminary Western blot studies comparing control and infected cells showed no notable differences in actin, vinculin, cortactin, α5 or β5 integrin expression, and immunofluorescence and confocal microscopy analyses were inconclusive (not shown).

### Extracellular BUNV in filament bundles of infected BHK-21 and MRC-5 cells

**Figure 5:**
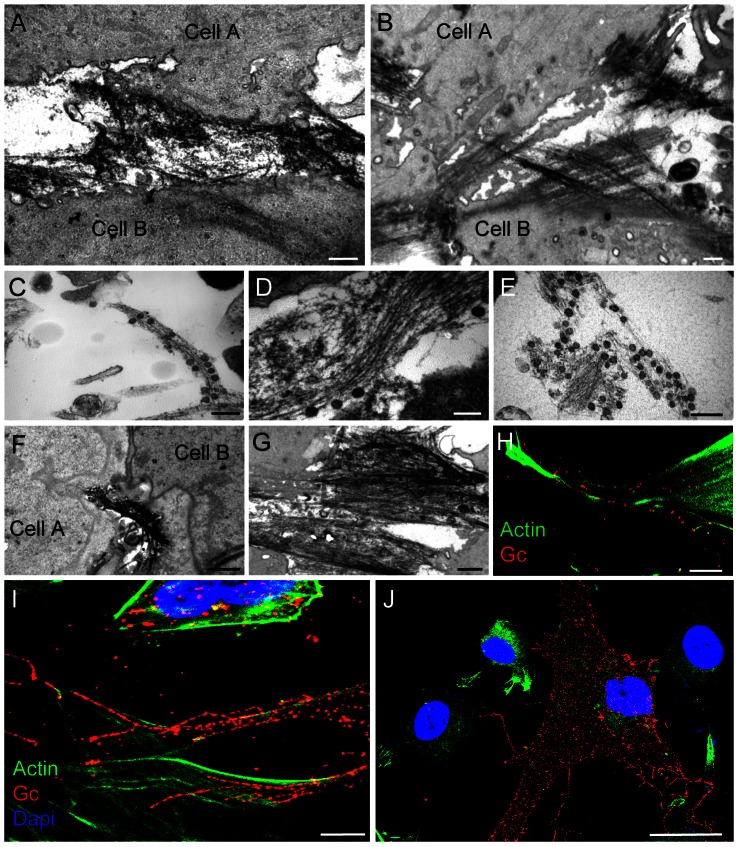
TEM and confocal microscopy of filament bundles on the basal surface of BUNV-infected cells. BHK-21 cells (A–E, H) and MRC-5 cells (F, G, I, J) are shown. (A, B) Filament bundles connecting neighboring BHK-21 cells. (C–E) TEM of various cells showing filament bundles with viral particles. (F, G) Dense filament bundles with viruses interconnecting MRC-5 cells. (H–J) Immunofluorescence and confocal microscopy of BUNV-infected cells, showing the distribution of actin (green; phalloidin) and viral particles (red; Alexa594-anti-Gc mAb); nuclei are DAPI-labeled (blue). Cells were labeled without prior permeabilization. Viral particles associate with extracellular bundles of actin filaments (H, I), which contact cells negative for anti-Gc and possibly uninfected (J). The infected cell in J (red) appears to contact four neighboring cells. Bars: 500 nm (A and B), 300 nm (C and E), 200 nm (D), 1 µm (F and G), 10 µm in H, 5 µm in I, 25 µm in J.

Oriented serial sectioning and TEM showed a second type of structure exclusive to cells that release BUNV virions from the basal surface (Figure 5). We observed dense filament arrays in the extracellular space, frequently connecting neighboring BHK-21 cells (Figure 5A, B); most extracellular virions were attached to these filaments (Figure 5C–E). Similar structures were found in the basal regions of BUNV-infected MRC-5 cells (Figure 5F, G) and, as for BHK-21 cells, these virus-bearing filaments frequently connected neighboring cells (Figure 5F). Given that BUNV exits the cell by active secretion, the association of newly formed viruses with the extracellular filament bundles is likely to occur outside the cell. Filament thickness and appearance suggested they could be made of cortical actin, and immunogold labeling with anti-actin antibodies and TEM confirmed that these extracellular filament arrays with viruses contain actin (Figure S4). Double labeling of actin and extracellular viruses followed by confocal microscopy confirmed that viral particles associated with actin filament bundles (Figure 5H, I) that connect infected with uninfected cells (Figure 5J). Like MLS, these filament arrays were best preserved when cells were fixed without removal of culture medium; they could only be visualized by TEM in oriented sections. TEM of BUNV-infected HEp-2 cells that release virus progeny through apical regions showed that these cells did not assemble dense extracellular filament arrays on basal regions; however, viruses released through the apical regions of HEp-2 cells were also surrounded by actin-like filaments (Figure 3H). These results point to a more general association of “extracellular” actin with Bunyavirus egress regardless of the surface used by the virus to exit the cell.

**Figure 6:**
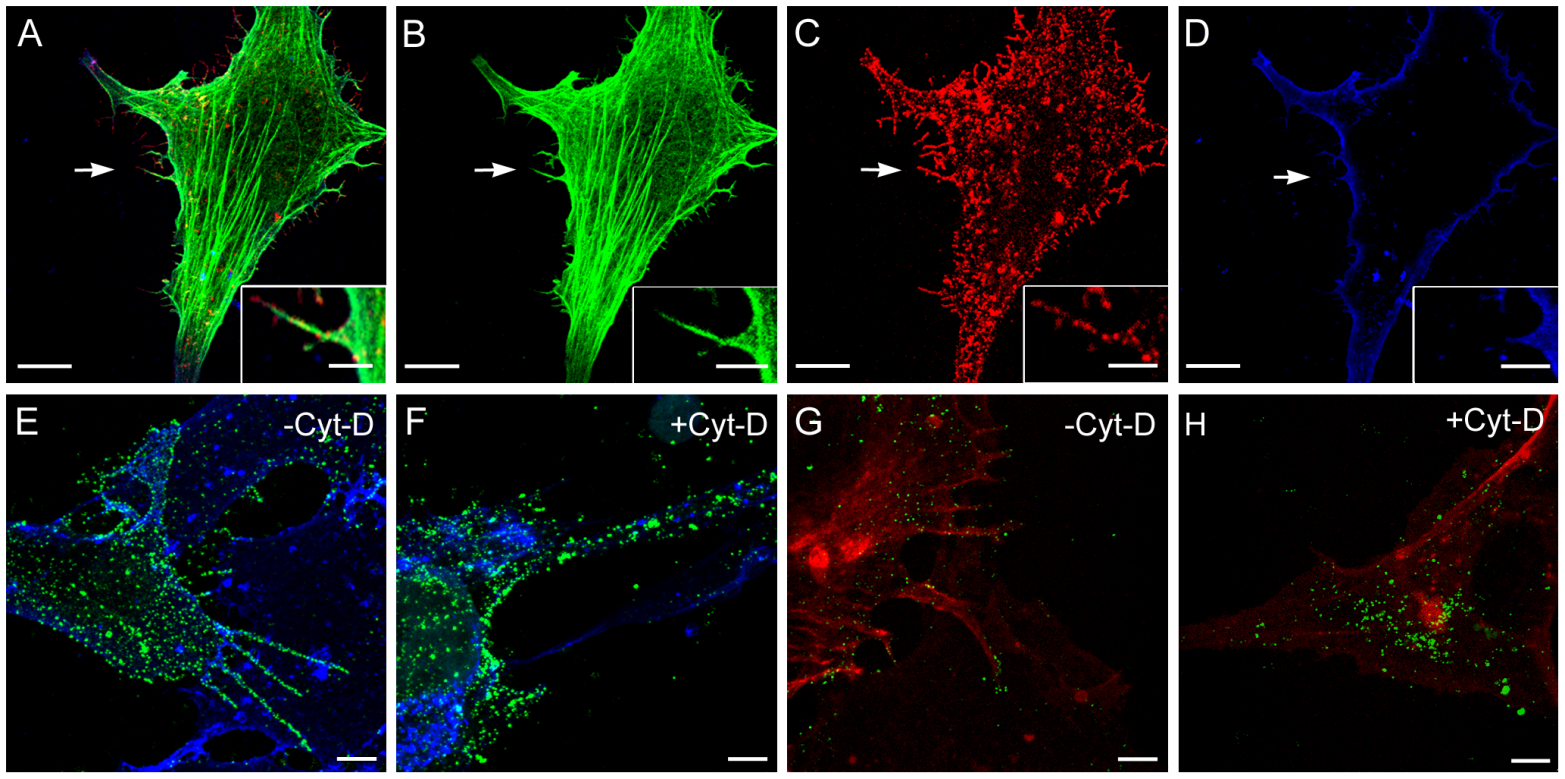
Association of BUNV and filaments on the cell surface and effects of cytochalasin D (Cyt-D). Non-permeabilized cells were labeled and visualized by confocal microscopy. (A–D) Confocal microscopy of an MRC-5 cell at 16 h.p.i. Actin was fluorescently labeled with phalloidin (green), virus particles with anti-Gc mAb (red) and plasma membrane with WGA-647 (blue). Merged (A) and separate images (B) and (C) show viruses and actin on cell surface projections (white arrows); no membrane appears to be associated to them (white arrow in D). Insets are enlargements of the cell surface projection marked with white arrows in main fields. (E–H) Confocal microscopy of BHK-21 cells infected alone (E, G) or with Cyt-D (F, H). (E) Viral particles (green) and plasma membrane (blue) at 10 h.p.i. (G) Actin (red) and virus (green) labeling, showing association of viral particles with actin-based projections on the cell surface. (F, H) Cyt-D-treated infected cells showing numerous viral particles (green) retained on the cell surface and bound to the plasma membrane (blue in F). In the presence of the drug, actin (red) no longer assembles filopodia or filament bundles (H). Bars: 10 µm (A, B, C and D), 3 µm (insets in A, B, C and D), 5 µm (E, F, G and H).

Extracellular viruses did not seem to attach to actin comets or filopodia, but rather to extracellular actin filament bundles that apparently lack a membrane (Figure 5). Examination of unpermeabilized cells labeled with phalloidin, anti-Gc and WGA to localize actin, virus and plasma membrane, respectively, supported this observation (Figure 6A–D). When MRC-5 cells infected at 1 PFU/cell and BHK-21 cells infected at 0.1 and 0.5 PFU/cell were treated at 1 h.p.i. with cytochalasin D (Cyt-D) to target actin, virus growth was mainly unaffected. In Cyt-D-treated cells, however, viral particles no longer associated with cell surface projections, but mainly remained attached to the plasma membrane (compare Figure 6E with 6F and Figure 6G with 6H). At 8 h.p.i., infectivity as titrated in culture supernatants decreased by two orders of magnitude in Cyt-D-treated compared to untreated cells (10^5^ vs 10^3^ PFU/ml); at 10 and 16 h.p.i., this decrease was only one order of magnitude. These data, and the morphological results shown in Figure 5, suggest that actin filaments might participate in BUNV release from infected cells, as well as in virus movement from cell to cell.

The extracellular actin bundles could facilitate propagation by transporting viruses to uninfected cells; using this railway, new viral particles would find and infect other cells more efficiently than if they were released and diluted in the culture medium. The function of these extracellular filament bundles thus resembles that of the actin comets induced by vaccinia or African swine fever virus [10], [38]–[41]. The structures identified here are nonetheless different; unlike actin comets, the filament arrays in BUNV-infected cells do not appear to use the plasma membrane for their assembly and function. However, we cannot exclude a participation of membranes in the assembly of filament bundles and that these membranes could have been dismantled at a later stage.

Extracellular actin filaments might also participate in virus egress in cells infected with the human immunodeficiency virus, HIV, in which similar extracellular filaments with attached viruses have been imaged [42]. The mechanisms of actin nucleation and polymerization in association with the plasma membrane have been well characterized, and our findings are likely to be debated. The role of these filaments in virus release is not yet fully understood and our TEM imaging studies of BUNV-infected cells only suggest that the extracellular filament bundles with attached viruses are not surrounded by a membrane or by membrane remnants. Further studies will be needed to determine their source and potential role in Bunyavirus infection.

### Conclusions

Our study identifies two previously unreported structures on the basal surface of two Bunyamwera virus-infected cell lines, consisting of multilamellar structures (MLS) and large extracellular bundles of actin filaments. Optimal preservation of cell basal surfaces followed by oriented sectioning before TEM was fundamental to the identification of these structures. This study also demonstrates the importance of CLEM as a strategy for selecting cells for analysis of fine details of the cell response to virus infection.

**Figure 7:**
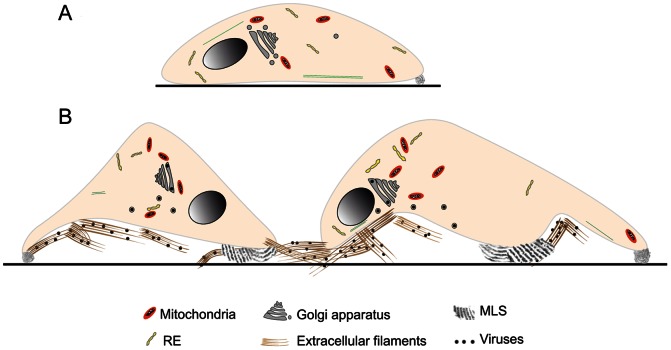
Scheme of our interpretation of the structures characterized in this work. (A) Control cell with membrane reservoirs, attached to the culture plate. (B) Infected cells with MLS and filament bundles with viruses. MLS could increase the ability of infected cells to attach to substrate; filament bundles might participate in cell-to-cell virus propagation.

In light of their morphology and dimensions, MLS are likely to be used by cells to increase their capacity to adhere to substrates, and the extracellular actin bundles might participate in virus propagation. We consider that during egress, viruses can induce assembly of filament bundles to cause partial detachment of cells from the substrate; cells would respond by organizing plasma membrane reservoirs into MLS to reinforce attachment, avoiding detachment and death. Our main findings and interpretation of the function of MLS and extracellular filaments in BUNV-infected cells is summarized in Figure 7.

We do not know whether the structures identified in this study have a role in *in vivo* virus infection, but it will be of interest to characterize their biogenesis. We speculate that MLS originate from cell surface membrane reservoirs, which are also used to assemble filopodia and lamellipodia. The potential origin of filament bundles is less clear; whether the filaments polymerize on the cell surface or are derived from the cytosol remains to be established. In future work, we will attempt to define factors involved in the biogenesis of MLS and filament bundles, and the role of extracellular matrix components in their assembly and function.

## Materials and Methods

### Cells, viruses, antibodies

BHK-21 (C13), MRC-5 (CCL-171) and HEp-2 (CCL-23) cell lines were supplied by the American Type Culture Collection (ATCC) and grown in Dulbecco's modified Eagle's medium supplemented with 10% fetal calf serum (Reactiva SA, Barcelona, Spain). BUNV (ATCCBR-87) was propagated in BHK-21 as described [23]. BUNV expressing EGFP-tagged NSm, rBUNM-NSm-EGFP [30], an antiserum to the extracellular Bunyamwera virions and the monoclonal antibody MAb-742 specific for BUNV Gc glycoprotein [43], [44] were kindly provided by Dr Richard M. Elliott (Centre for Biomolecular Sciences, University of St. Andrews, UK). Rabbit anti-NC antiserum was obtained by immunization with a synthetic peptide corresponding to the amino terminal region of the protein [22]. Anti-β actin mAb AC-15 and anti-vinculin mAb hVIN-1 were from Sigma-Aldrich, anti-cortactin (p80/85) 4F11 from Millipore, and anti-CD63 mAb from Immunostep. Polyclonal anti-α5-integrin antibody (ab55991) and anti-β5-integrin mAb (ab55359) were from Abcam. Rabbit anti-Lamp1 was kindly provided by Dr Sven R. Carlsson (Umeå University, Sweden). Fluorescent secondary antibodies (conjugated with Alexa488 or Alexa594), phalloidin (Alexa488 or Alexa594), fluorescent wheat germ agglutinin (WGA with Alexa594 or Alexa647) were from Molecular Probes, the DAPI nuclear marker from Sigma-Aldrich, the Lysotracker RED (DND-99) from Invitrogen and secondary antibodies conjugated with colloidal gold particles from BB International.

### Infections and treatments with trypsin-EDTA and cytochalasin D

Monolayers of BHK-21 or MRC-5 cells were infected with BUNV at a multiplicity of infection (M.O.I.) of 1 plaque-forming unit (PFU) per cell. At 8, 10, 12, 14 and 16 hours post-infection (h.p.i.), culture supernatants were removed and cell monolayers processed for embedding and TEM. BHK-21 cells were titrated by plaque assay [24]; MRC-5 cells were titrated by immunofluorescence using anti-BUNV polyclonal antiserum. HEp-2 cell monolayers were infected with BUNV or rBUNM-NSm-EGFP recombinant virus at 1 PFU/cell and processed at 8, 14, 18, 24, 30 and 48 h.p.i. Supernatants were titrated by plaque assay using BHK-21 cell monolayers.

For trypsin-EDTA treatment, BHK-21, MRC-5 and HEp-2 cells were grown on M-24 culture plates; 80% confluent monolayers were infected with BUNV at 1 PFU/cell for 10 h (BHK-21 cells) 16 h (MRC-5 cells) or 24 h (HEp-2 cells). Cells were then incubated (37°C) with 0.25% (w/v) trypsin and 0.2% (w/v) EDTA in PBS. Cells that remained attached to culture plates were counted at 0, 1, 2, 5, and 10 min after replacing culture medium with trypsin-EDTA. Cells were counted in a Zeiss Axiovert 40–CFL light microscope.

For treatment with the actin-disrupting drug cytochalasin D (Cyt-D; Sigma-Aldrich), MRC-5 cells were infected with BUNV at 1 PFU/cell and BHK-21 cells at 0.1 or 0.5 PFU/cell; at 1 h.p.i., culture medium was removed and replaced with medium containing 2 mM Cyt-D. Aliquots were collected at 8, 10 and 16 h.p.i. and processed for virus titration, while cell monolayers were fixed without previous washing and processed for immunolabeling and fluorescence microscopy.

### Immunofluorescence, confocal, live cell video, and differential interference contrast (DIC) light microscopy

Samples were prepared for immunofluorescence as described [45]. To label the filament bundles on the cell surface, we used a “cytoskeleton buffer” without saponin (10 mM morpholineethanesulfonic acid (MES), 150 mM NaCl, 5 mM MgCl_2_, 5 mM EGTA, and 5 mM glucose, pH 6.1). Fluorescent WGA-594 (red) or WGA-647 (blue) were used to label the plasma membrane without previous permeabilization. Images were collected in a Leica TCS SP5 confocal microscope.

For live cell video microscopy, BHK-21 cells were cultured on glass-bottom dishes (P35G-1.5-20-C; MatTek). Cell monolayers were infected with BUNV at 1 PFU/cell in the presence of fluorescent WGA-594 (1∶200) in culture medium. WGA-594 fluorescence and DIC images were collected every 15 min from 0 to 18 h.p.i. Digital photographs were processed with Adobe Photoshop, ImageJ, LAS and CW4000 FISH (Leica Microsystems) software.

### Correlative light and electron microscopy (CLEM)

For CLEM studies, BHK-21 cell monolayers were cultured on gridded Thermanox plastic coverslips (Nunc) and infected with BUNV at a M.O.I. of 1 PFU/cell. At 10 h.p.i., cells were fixed without removing the culture medium and processed for embedding and TEM. Cells with interesting features were selected in a Zeiss Axiovert 40–CFL light microscope, dehydrated and embedded in EML-812 epoxy resin (Taab Laboratories) [22]. After resin polymerization, preselected cells were localized in the first ultra-thin (∼70 nm) sections; these were obtained in a Leica EM UC6 ultramicrotome, collected on formvar-coated copper grids and stained with uranyl acetate and lead citrate.

For CLEM studies with HEp-2 cells, monolayers were cultured on gridded glass-bottom plates (P35G-2-14-CGRD; MatTek). Cells were infected with recombinant rBUNM-NSm-EGFP virus (M.O.I. 1 PFU/cell); at 30 h.p.i., cells were fixed and embedded in LR White acrylic resin (London Resin Company Ltd) [46]. Infected fluorescent cells were selected in a Leica TCS SP5 confocal microscope and fixed with a mixture of 4% paraformaldehyde (PFA) and 0.1% glutaraldehyde (4°C, 1 h). After washing with PBS, cell monolayers were dehydrated with ethanol, infiltrated with resin and polymerized. Resin-embedded cell monolayers were separated from glass coverslips by repeated immersion in liquid nitrogen and warm water. Ultrathin sections were collected on formvar-coated 50-GP copper grids and stained with uranyl acetate and lead citrate. All samples were studied in a Jeol JEM 1011 electron microscope operating at 100 kV.

### Oriented serial sections, TEM and 3D reconstructions

Consecutive ultrathin (∼70 nm) sections were collected on formvar-coated parallel-bar copper grids, stained with uranyl acetate and lead citrate, and studied by TEM. A total of 41 complete series were stained and studied; three of these (5–6 sections each) were subsequently processed for 3D reconstruction. Photographs of regions of interest in the series were taken at a nominal magnification of 6,000× and 10,000×. Plates were digitized as 8-bit images, 3.39 nm final pixel size and 600 dpi resolution using an Epson Perfection Photo 3170 scanner. Images were then normalized using Bsoft software [47]; (http://lsbr.niams.nih.gov/bsoft/). Digital images were aligned by selected tracers between two consecutive sections using the free editor for serial section microscopy ‘Reconstruct’ [48]; (http://www.synapse-web.org/tools/index.stm/). Segmentation and 3D visualization were done with Amira (http://amira.zib.de) [22].

### Immunoelectron microscopy

For immunogold labeling studies, ultrathin sections of cells embedded in LR White acrylic resin were incubated (30 min) with Tris buffer-gelatin (TBG, 30 mM Tris-HCl, pH 8.0, with 150 mM NaCl, 0.1% bovine serum albumin, 1% gelatin) and with primary antibodies diluted in TBG (1∶200, anti-BUNV N; 1∶100, anti-actin). After washing, sections were incubated 1 h with a secondary antibody conjugated with 10 or 15 nm colloidal gold particles and diluted 1∶40 in TBG. Grids were allowed to dry before staining with saturated uranyl acetate and studied by electron microscopy.

## Supporting Information

Figure S1 **Scheme summarizing the principles of oriented embedding and sectioning of cell monolayers for TEM.** Cells are cultured on plastic Thermanox coverslips and embedded in epoxy resin. Cell monolayers are mounted for ultramicrotomy to obtain serial sections oriented parallel (left) or perpendicular (right) to the cell base; sections are then collected on EM grids, stained, and studied in a transmission electron microscope.
(TIF)

Figure S2 **3D TEM of an MLS from a BUNV-infected BHK-21 cell.** Ultrathin serial sections (A–E) and 3D reconstruction (F). Connections between the MLS and plasma membrane were clearly detected in some individual planes (arrows in A, C) and in the 3D volume (F). Bars: 200 nm.
(TIF)

Figure S3 **Confocal microscopy of Lysotracker- and WGA-labeled BHK-21 cells.** Control (A) and BUNV-infected cells at 14 h.p.i. (B). At this t.p.i. and a MOI of 1 PFU/cell, all cells in the monolayer were infected. Cells were labeled without permeabilization. Images on the bottom (A1 to A9 and B1 to B9) are Z series from the frontal projections shown in (A) and (B). For each image the distance from the adherent surface is indicated. Bars: 25 µm.
(TIF)

Figure S4 **Immunogold labeling and TEM of filament bundles on the basal surfaces of BUNV-infected cells.** Ultra-thin sections of BUNV-infected BHK-21 (A, B) and MRC-5 cells (C, D), labeled at 16 h.p.i. with anti-actin mAb followed by a secondary antibody conjugated with 10 or 15 nm colloidal gold particles (black arrowheads). Labeling concentrates in the extracellular filament bundles with viral particles (white arrowheads). Bars: 100 nm (A, C and D), 50 nm (B).
(TIF)
