# Supplementary figures and images for: Multilamellar Structures and Filament Bundles Are Found on the Cell Surface during Bunyavirus Egress
Source: PLoS One. 2013 Jun 14;8(6):e65526. doi: 10.1371/journal.pone.0065526 (PMC3683019; doi:10.1371/journal.pone.0065526)

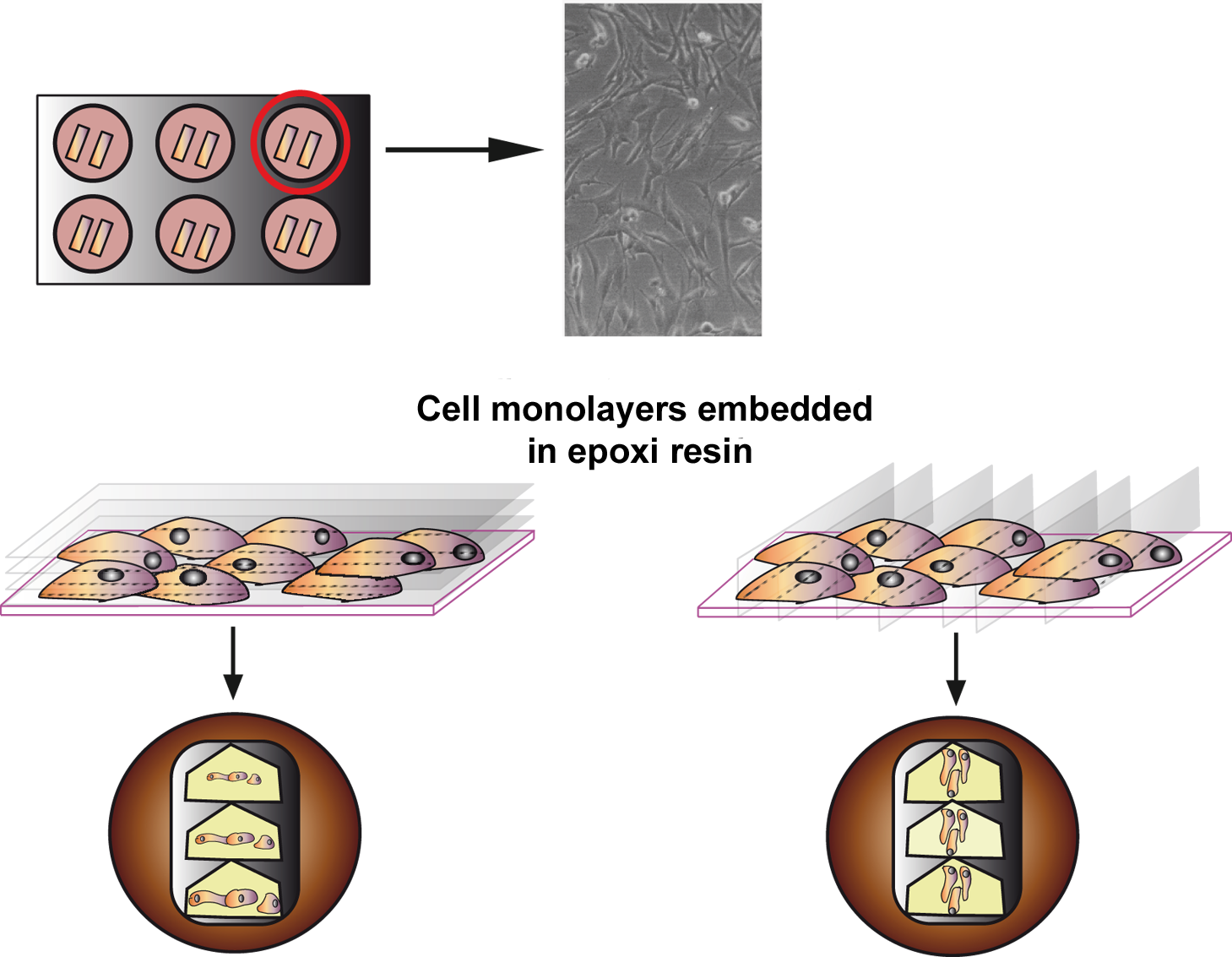

Supplement: Figure S1 — Scheme summarizing the principles of oriented embedding and sectioning of cell monolayers for TEM. Cells are cultured on plastic Thermanox coverslips and embedded in epoxy resin. Cell monolayers are mounted for ultramicrotomy to obtain serial sections oriented parallel (left) or perpendicular (right) to the cell base; sections are then collected on EM grids, stained, and studied in a transmission electron microscope. (TIF) [file pone.0065526.s001.tif]

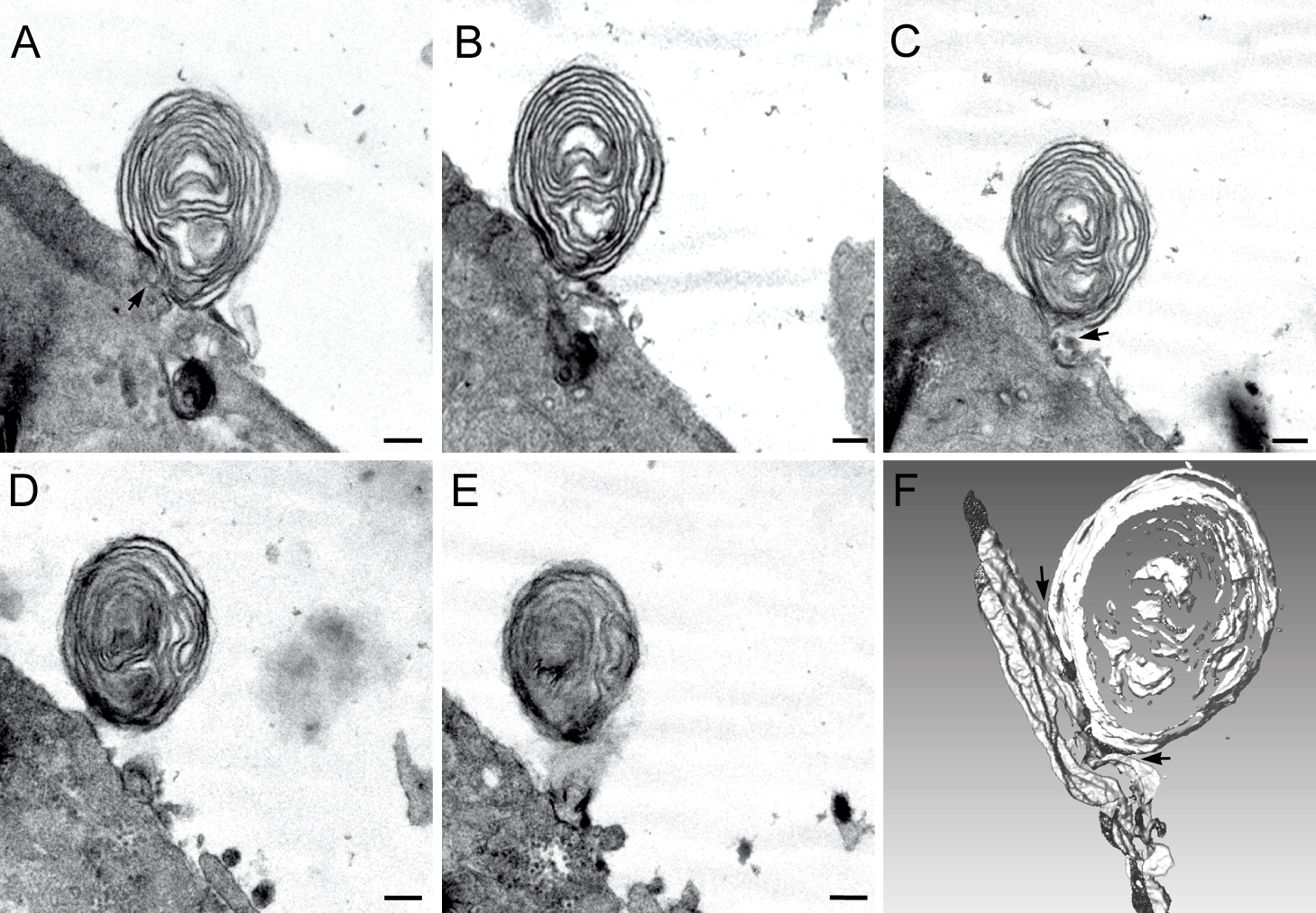

Supplement: Figure S2 — 3D TEM of an MLS from a BUNV-infected BHK-21 cell. Ultrathin serial sections (A–E) and 3D reconstruction (F). Connections between the MLS and plasma membrane were clearly detected in some individual planes (arrows in A, C) and in the 3D volume (F). Bars: 200 nm. (TIF) [file pone.0065526.s002.tif]

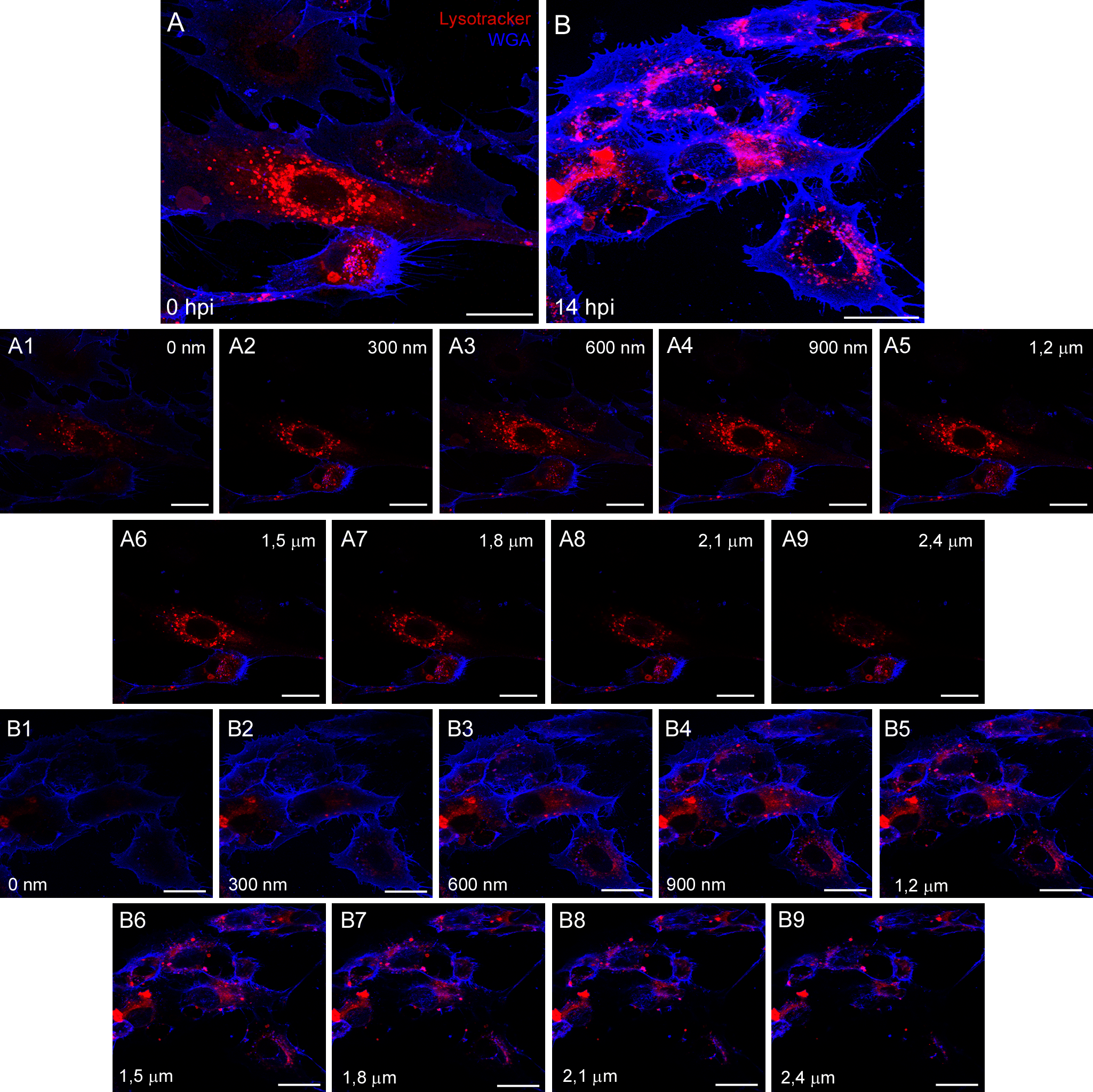

Supplement: Figure S3 — Confocal microscopy of Lysotracker- and WGA-labeled BHK-21 cells. Control (A) and BUNV-infected cells at 14 h.p.i. (B). At this t.p.i. and a MOI of 1 PFU/cell, all cells in the monolayer were infected. Cells were labeled without permeabilization. Images on the bottom (A1 to A9 and B1 to B9) are Z series from the frontal projections shown in (A) and (B). For each image the distance from the adherent surface is indicated. Bars: 25 µm. (TIF) [file pone.0065526.s003.tif]

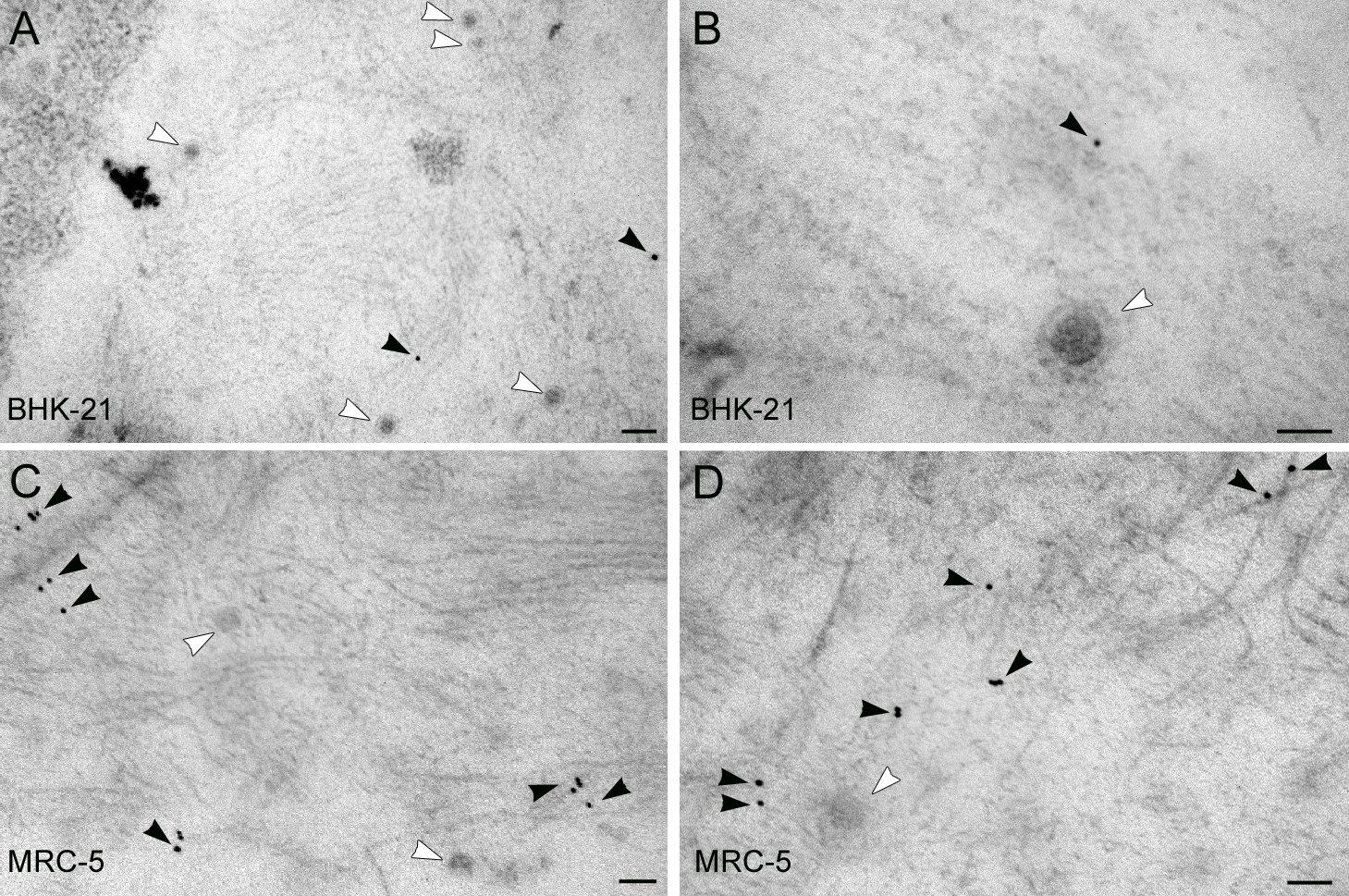

Supplement: Figure S4 — Immunogold labeling and TEM of filament bundles on the basal surfaces of BUNV-infected cells. Ultra-thin sections of BUNV-infected BHK-21 (A, B) and MRC-5 cells (C, D), labeled at 16 h.p.i. with anti-actin mAb followed by a secondary antibody conjugated with 10 or 15 nm colloidal gold particles (black arrowheads). Labeling concentrates in the extracellular filament bundles with viral particles (white arrowheads). Bars: 100 nm (A, C and D), 50 nm (B). (TIF) [file pone.0065526.s004.tif]
